# How phytoplankton compete for nutrients despite vast intercellular separation

**DOI:** 10.1093/ismeco/ycae003

**Published:** 2024-01-18

**Authors:** Ben A Ward

**Affiliations:** School of Ocean and Earth Science, University of Southampton, European Way, Southampton, UK

**Keywords:** plankton, nutrients, competition, diffusion, uptake, drawdown

Phytoplankton are the most abundant photosynthetic organisms on Earth, with the smallest cells reaching concentrations, $P$, on the order of 100 billion cells per cubic metre [1]. Nonetheless, even at such high population densities, the smallest phytoplankton are extremely sparsely distributed on the scale of individuals. If we consider the average amount of water inhabited by a single cell being equal to the inverse of the cellular concentration, $P^{-1}$ [2], we find a volume of 10 million ${\mu }m^{3}$. This is about 100 million times larger than the typical volume of a *Prochlorococcus* cell—a size ratio roughly equivalent to a single ping pong ball in an Olympic swimming pool. Given the enormous relative distances between individual cells, some authors have questioned whether it is reasonable to assume that phytoplankton can compete for nutrients [2–4].

To illustrate this reasoning, we can consider a single spherical cell of radius $R$. It is often assumed that cellular uptake leads to depletion of nutrients at the cell surface, relative to the general medium [2]. This creates a gradient in the nutrient field around the cell, with nutrient concentrations increasing with distance from the cell (Fig. 1). Seeking to quantify the magnitude of this so-called boundary layer, Siegel [2] defined a cellular “sphere of influence” as the region within which a cell could deplete nutrients to less than 80% of the concentration far from the cell. Regardless of cell size, this sphere of influence turns out to be five times the cell diameter (Fig. 1).

**Figure 1:**
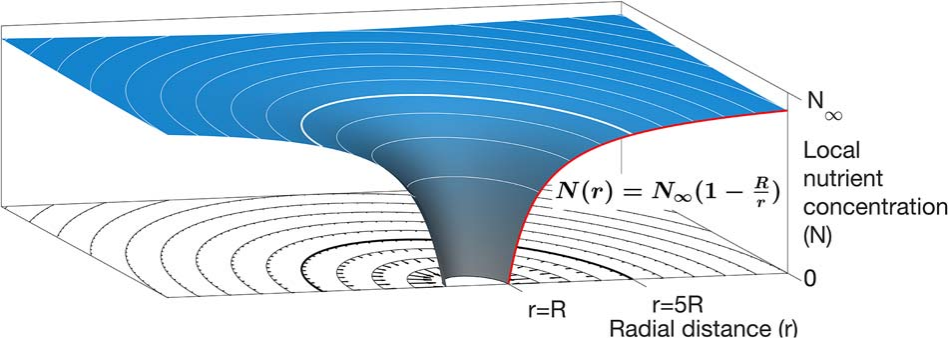
The diffusive boundary layer around a two-dimensional representation of a spherical cell. Notes: The figure shows how the diffusive flux of nutrients towards the cell is theoretically maintained by a gradient in the resource concentration. For a cell of radius $R$, the local nutrient concentration is shown by the height of the blue surface. Diffusive fluxes towards the cell (arrows) are proportional to the nutrient gradient. If nutrients are drawn down completely at the cell surface, the concentration at radius $r$ outside the cell is given by $N(r) = N_{\infty }(1-{R}/{r})$ (where *N*_∞_ is nutrient concentration far from the cell [2]). The plotted circles show distances at integer multiples of $R$. The extent of the sphere of influence ($r=5R$; [2]) is shown by the thicker lines.

Using the cell densities defined above, *Prochlorococcus* cells are separated by an average distance of 400 times their cellular diameter—vastly greater than the extent of their assumed spheres of influence. This raises the question, if the spatial distancing of phytoplankton is so large that boundary layers cannot overlap, how can phytoplankton compete for nutrients [4]?

In the following I will show that the size of the nutrient boundary layer is irrelevant when considering the ability of phytoplankton to deplete nutrient concentrations at the ocean surface—it is not the size of the boundary that determines nutrient uptake, but rather the rate at which nutrients flow through it.

As an intuitive analogy, we can consider the way water levels within a large reservoir can be effectively regulated by a relatively tiny drainage spillway. Fig. 2A shows water flowing through the drainage spillway of Lake Berryessa in California. As is the case for the diffusion of nutrients towards a cell, the spillway has a clear boundary layer, beyond which water levels appear to be relatively unaffected by the presence of the drain.

**Figure 2:**
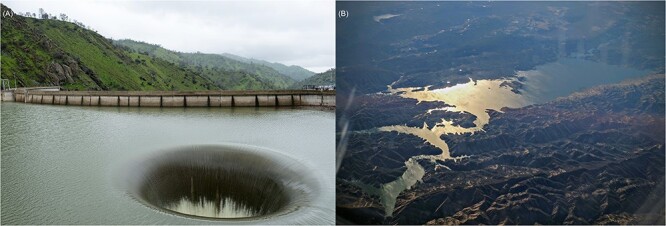
The “Glory Hole” spillway (A) at Lake Berryessa (B). Notes: The drainage spillway and dam are located near the bottom left of the aerial picture. Image credit: left, “Monticello Dam spillway, Lake Berryessa” by Jeremy Brooks; right, “Aerial view of Lake Berryessa” by Dick Lyon. Both images can be found on commons.wikimedia.org, and are licensed under CC BY-SA 4.0 (https://creativecommons.org/licenses/by-sa/4.0/).

At its widest point, the Lake Berryessa spillway has a diameter of only 22 m. It is, however, reasonable to expect that the spillway can regulate excess water levels across the entire 80 million m$^{2}$ area of the lake (Fig. 2B). This is because there is a large flow of water through the spillway over time. Indeed, with an estimated maximum flow rate of $\sim $1400 m$^{3}$ s$^{-1}$, the spillway has the capacity to lower water levels across the entire area of the lake by 1 m in just 16 hours.

Phytoplankton cells are similarly able to deplete nutrients over wide areas of the ocean, despite very large intracellular distances. This is possible because molecular diffusion can sustain very rapid fluxes through their boundary layers [5]. This can be seen if we assume that the diffusive uptake of some generic nutrient $N$ is equal to the diffusive flux through the boundary layer of a single cell ($4\pi R \kappa N$; [2]), multiplied by the population abundance, $P$ (Here $ \kappa $ is the molecular diffusivity of the nutrient ions in seawater). For a population of a given cell size and abundance, unrestricted uptake by diffusion would therefore remove nutrient from the water at a rate of of $dN/dt = -4 \pi R \kappa P N$. From this, the characteristic time scale of nutrient depletion can be expressed as the time taken for this flux to decrease the nutrient concentration by half, $t_{1/2} = \ln ({2})(4 \pi R \kappa P)^{-1}$.

Fig. 3 shows how this theoretical time scale varies as a function of cell size and abundance. For observed *in situ* abundances [6], it can be seen that diffusion-limited uptake by the smallest cells can remove nutrients from the water extremely quickly. Indeed, in the absence of any nutrient supply by mixing or remineralization, a population of 0.6 ${\mu }m$ diameter *Prochlorococcus* cells at an abundance of 10$^{11}$ cells m$^{-3}$ has a theoretical capacity to halve the environmental nutrient concentration in just 22 minutes. Even cells as large as 20 ${\mu }m$ in diameter are associated with time scales of the order of just days, when present in sufficient abundance.

**Figure 3:**
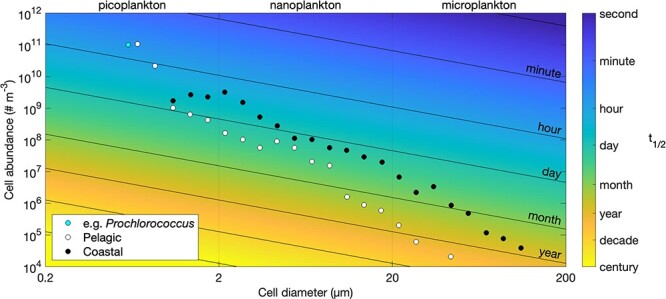
Time taken for diffusion-limited uptake to decrease any given ambient nutrient concentration by half ($t_{1/2}$), as a function of cell size and abundance. Notes: The blue dot shows the size and abundance of the *Prochlorococcus* population used as an example in the main text. The black and white dots show observed sizes and abundances from coastal and pelagic regions of the North Atlantic [6]. Time scales are shorter at the coastal site because there are more cells in each size class to take up nutrients. Here the molecular diffusivity, $\kappa $, is assigned a value of $1.4\times 10^{-9}$ m$^{2}$ s$^{-1}$, which is representative of a range of nutrient ions in seawater [2].

In practice, such rapid uptake will be moderated by other limiting factors, including cross-membrane transport and metabolism, while the very slow diffusive uptake among larger cells may be enhanced by morphological adaptations and turbulent diffusion. Nonetheless, the longer time scales of nutrient drawdown associated with larger cells illustrate how larger phytoplankton struggle to compete when nutrients are very rapidly removed from the water by smaller cells.

These calculations demonstrate that the small sizes of diffusive boundary layers present no theoretical barrier to rapid depletion of nutrients by phytoplankton. Nutrient depletion is also unequivocally observed both in laboratory cultures [7] and in the ocean during phytoplankton blooms [8], and uptake by phytoplankton is the only available explanation for the observed low nutrient concentrations across the vast areas of the ocean’s subtropical gyres.

The arguments presented above highlight an important distinction between two forms of competition in ecology, namely interference and exploitation competition [9]. Interference competition occurs when organisms interact directly to compete for a limiting resource. For example, the presence of a tree in a forest prevents another tree from occupying the same space [4]. Exploitation competition, however, occurs when individuals respond to the availability of a resource that has been depleted by the activity of other individuals [9]. These individuals do not need to ever directly interact. It is only required that they affect, and are affected by, the availability of a shared resource in the same general environment.

The theoretical concept of the nutrient boundary layer demonstrates that interference competition is often unlikely in the vast three-dimensional space of the ocean [2–4]. However, while the diffusive boundary layer may be relatively small, the rapid uptake of nutrients through time (accompanied by population growth and the subsequent export of organic matter) can very efficiently strip water of nutrients, allowing exploitation competition [9].

Nutrient uptake needs to be understood in terms of its time-dependent influence on overall nutrient concentrations, not through its static and time-independent ability to develop nutrient gradients in very localized regions around the cell. Intercellular distances corresponding to hundreds of body lengths may seem incompatible with nutrient drawdown and competition, especially when considered with respect to the size of the theoretical diffusive boundary layer. However, when we consider the rate at which nutrient ions diffuse at these tiny scales [5], combined with there being no need for direct interactions in the context of exploitation competition, we see that we do not need to discard the well-established concept of resource competition among the plankton.

As a final comment, none of the ideas presented above should be interpreted as evidence that resource competition is the single dominant factor regulating the biodiversity and structure of marine microbial communities, or that we should always expect competitive exclusion. In his formative essay, Hutchinson [10] identified several mechanisms by which phytoplankton species can coexist in the complex marine environment, including non-equilibrium dynamics and top-down control by predators. Species may also coexist indefinitely if they are ecologically neutral (i.e. they show an effectively identical fitness in their environment; [4]). None of these mechanisms is incompatible with resource competition. A key challenge is therefore to understand and quantify how the balance of these different mechanisms shifts through time and space and across different trophic levels to shape the structure and function of marine communities.

## Data Availability

The data used in Fig. 3 were extracted from figure 1 in Marañón [6].
